# *Ophiophagus hannah* Venom: Proteome, Components Bound by *Naja kaouthia* Antivenin and Neutralization by *N. kaouthia* Neurotoxin-Specific Human ScFv

**DOI:** 10.3390/toxins6051526

**Published:** 2014-05-13

**Authors:** Witchuda Danpaiboon, Onrapak Reamtong, Nitat Sookrung, Watee Seesuay, Yuwaporn Sakolvaree, Jeeraphong Thanongsaksrikul, Fonthip Dong-din-on, Potjanee Srimanote, Kanyarat Thueng-in, Wanpen Chaicumpa

**Affiliations:** 1 Graduate Program in Immunology, Faculty of Medicine Siriraj Hospital, Mahidol University, Bangkok 10700, Thailand; E-Mail: witchu.joy@hotmail.com; 2 Department of Molecular Tropical Medicine and Genetics, Faculty of Tropical Medicine, Mahidol University, Bangkok 10400, Thailand; E-Mail: onrapak.rea@mahidol.ac.th; 3 Department of Research and Development, Faculty of Medicine Siriraj Hospital, Mahidol University, Bangkok 10700, Thailand; E-Mail: nitat.soo@mahidol.ac.th; 4 Laboratory for Research and Technology Development, Department of Parasitology, Faculty of Medicine Siriraj Hospital, Mahidol University, Bangkok 10700, Thailand; E-Mails: watee.see@gmail.com (W.S.); jub922@yahoo.com (Y.S.); gskmu@hotmail.com (J.T.); mam_mt41@hotmail.com (K.T.); 5 Graduate Program in Biomedical Science, Faculty of Allied Health Sciences, Thammasat University, Pathumthani 12120, Thailand; E-Mail: srimanote_p@y7mail.com; 6 Center for Agriculture Biotechnology and Department of Veterinary Pathology, Faculty of Veterinary Medicine, Kasetsart University, Kam-paeng-saen Campus, Nakhon-pathom 73140, Thailand; E-Mail: fddvt66@outlook.com; 7 Department of Microbiology and Immunology, Faculty of Veterinary Medicine, Kasetsart University, Bangkok 10900, Thailand

**Keywords:** antivenin, human ScFv (HuScFv), paraspecificity, *Naja kaouthia*, *Ophiophagus hannah*, proteome

## Abstract

Venomous snakebites are an important health problem in tropical and subtropical countries. King cobra (*Ophiophagus* *hannah*) is the largest venomous snake found in South and Southeast Asia. In this study, the *O. hannah* venom proteome and the venom components cross-reactive to *N. kaouthia* monospecific antivenin were studied. *O. hannah* venom consisted of 14 different protein families, including three finger toxins, phospholipases, cysteine-rich secretory proteins, cobra venom factor, muscarinic toxin, L-amino acid oxidase, hypothetical proteins, low cysteine protein, phosphodiesterase, proteases, vespryn toxin, Kunitz, growth factor activators and others (coagulation factor, endonuclease, 5’-nucleotidase). *N. kaouthia* antivenin recognized several functionally different *O. hannah* venom proteins and mediated paratherapeutic efficacy by rescuing the *O. hannah* envenomed mice from lethality. An engineered human ScFv specific to *N. kaouthia* long neurotoxin (NkLN-HuScFv) cross-neutralized the *O. hannah* venom and extricated the *O. hannah* envenomed mice from death in a dose escalation manner. Homology modeling and molecular docking revealed that NkLN-HuScFv interacted with residues in loops 2 and 3 of the neurotoxins of both snake species, which are important for neuronal acetylcholine receptor binding. The data of this study are useful for snakebite treatment when and where the polyspecific antivenin is not available. Because the supply of horse-derived antivenin is limited and the preparation may cause some adverse effects in recipients, a cocktail of recombinant human ScFvs for various toxic venom components shared by different venomous snakes, exemplified by the *in vitro* produced NkLN-HuScFv in this study, should contribute to a possible future route for an improved alternative to the antivenins.

## 1. Introduction

Snake envenomation is an important health problem and occupational hazard among outdoor workers, such as farmers, plantation workers and agricultural harvesters, in tropical and subtropical areas, where venomous snakes have a habitat predilection [1,2,3]. It has been estimated that more than 50,000 deaths occur yearly from the snakebites [3]. The majority of cases were from rural areas where access to healthcare facilities is limited and antivenins are usually not available [1,2,3,4,5]. The treatment mainstay of the venomous snakebites in Thailand relies on the horse-derived antivenins produced by Queen Saovabah Memorial Institute (QSMI), Bangkok. The antivenins may be either monospecific for cases when the causative snakes are known or polyspecific, which neutralizes more than one venom species when biting snakes are not identified [5]. The therapeutic efficacy of the latter depends highly on the amounts of the antibodies that could neutralize the heterologous causative venom components (paraspecificity) [6,7]. Therefore, insight into the venom proteomes of individual venomous snake species that inhabit common geographical areas/localities, like *O. hannah* and *N. kaouthia*, which produce similar clinical features [7,8] and the identification of common components shared among their venoms should be useful information for treatment indication when homologous and polyspecific antivenins are not available. In this study, the *O. hannah* venom proteome was characterized, and the components cross-reactive to horse derived-monospecific *N. kaouthia* antivenin were determined. Paraspecific immunity mediated by the antivenin was evaluated also in mice.

Basically, treatment with the horse-derived antivenins is highly effective. Nevertheless, there have been limitations in the production, supply and use of the remedies. The production of immune sera in large animals requires adequate and appropriate animal husbandry, including pasture for grazing, shelter, an animal care taker and a veterinarian. For immunization, the snake venom or a mixture of venoms of several snake species in the immunological adjuvant is injected into the animal at multiple sites. Several boosters are required over an extended period of time (6–12 months or longer) in order to expect the satisfactory serum antibody levels. The quality of the antivenin is subjected to batch-to-batch/animal-to-animal variation. The amount of immune globulin obtained from individual animals at each bleeding time is limited. As such, in some regions of the world where antivenin is not available, many snake bitten subjects receive only traditional panaceas and/or palliative treatment. Besides, the preparations also contain a large fraction of nonspecific proteins. Thus, the antivenin dosage for the treatment of snakebites has never been certain and can be based only on the degree of envenomation [9]. Approximately 20% of the recipients develop either immediate hypersensitivity, including allergy and anaphylaxis and/or late serum sickness, due to the human anti-animal isotype response [6]. Although the early adverse reactions are readily managed in clinical settings with the use of adrenaline, anti-histamines and steroids, the late anti-animal isotype response is difficult to avoid. With contemporary technology, such as the phage display technique, the production of standardized recombinant antibodies to any required target antigen is possible *in vitro* without the prolonged animal immunization process and *in vivo* immune regulations by using the antibody phage display library as a biological tool [10].

Recently, human single-chain variable antibody fragments (HuScFv) specific to *N. kaouthia* long neurotoxin (NkLN-HuScFv) were produced *in vitro* [10]. The engineered HuScFv could rescue the *N. kaouthia* envenomized mice from lethality. Moreover, humanized-camel single-domain antibodies (sdAb) specific to *N. kaouthia* phospholipase A2 (PLA_2_) prepared from a humanized-camel VH/V_H_H (nanobody) phage display library have been shown to neutralize the enzymatic activity of the detrimental enzyme [11]. Therefore, it is envisioned that a cocktail of human/humanized-small antibodies, which are devoid of Fc fragments (thus, not causing an additional inflammatory response) and specific to venomous components should be a possible future road for anti-snake venom design. In this study, the ability of NkLN-HuScFv in rescuing the *O. hannah* envenomed mice from lethality was determined as an example of recombinant-specific antibodies that can mediate paraspecificity.

## 2. Materials and Methods

### 2.1. Animals

Male Institute of Cancer Research (ICR) mice, 5 weeks old, were from The National Laboratory Animal Center, Mahidol University, Nakhonpathom, Thailand. Animal husbandry and manipulation were performed following the guideline of the National Research Council of Thailand. Animal experiments were approved by the Siriraj Animal Care and Use Committee (SiACUC), Faculty of Medicine, Siriraj Hospital, Mahidol University (COA No. 004/2556).

### 2.2. O. hannah Venom, Horse-Derived N. kaouthia Antivenin and HuScFv-Specific to N. kaouthia Long Neurotoxin (NkLN-HuScFv)

*O. hannah* holovenom and horse-derived monospecific *N. kaouthia* antivenin (purified equine F(ab)’2) were obtained in lyophilized form from the Queen Saovabah Memorial Institute (QSMI). The lyophilized antivenin was dissolved in ten mL of ultrapure sterile distilled water (UDW), while the venom was dissolved in one mL of normal saline solution (NSS). Protein concentrations of the preparations were determined by using Bradford’s reagent (Bio-Rad, Hercules, CA, USA).

For preparing NkLN-HuScFv, the gene sequence coding for the HuScFv (*huscfv*) in phagemid transformed *E. coli* clone no. P8/22/3 [10] was subcloned into pET23b^+^, and the recombinant plasmids were put into BL21 (DE3) *E. coli*. The transformed bacteria were grown under IPTG induction condition, and soluble NkLN-HuScFv was purified from the bacterial lysate by using Ni-NTA affinity resin (Thermoscience, Rockford, IL, USA). The *E. coli-*derived NkLN-HuScFv has been shown to neutralize *N. kaouthia* neurotoxin and rescued the *N. kaouthia* envenomed mice from lethality [10]. By using the phage peptide mimotope search and multiple alignments, the HuScFv was found to bind to amino acids in loop 3 of *N. kaouthia* long neurotoxin (accession No. 229777), which is the venom binding site to the neuronal acetylcholine receptor (AchR) [10].

### 2.3. Characterization *of* O. hannah Venom Proteome by 1DE-ESI-LC-MS/MS

*O. hannah* venom was denatured by heating at 95 °C for 5 min in sample buffer (60 mM Tris-HCl, pH 6.8, 2% (w/v) SDS, 10% (v/v) glycerol, 1% (v/v) β-mercaptoethanol and bromophenol blue). The sample was subjected to 12% SDS-PAGE and stained with Coomassie Brilliant Blue R-250 (CBB) dye. The SDS-PAGE gel was cut horizontally into 10 equal pieces, destained in 100 μL of 50% (v/v) acetonitrile in ammonium bicarbonate and 100 µL of 4 mM dithiothreitol (DTT), kept at 60 °C for 15 min, alkylated by adding 7 µL of 250 mM iodoacetamide and kept in the dark for 40 min. Excess iodoacetamide was quenched with 3 µL of 4 mM dithiothreitol (DTT). All preparations were dehydrated by using acetonitrile, rehydrated with trypsin solution and incubated at 37 °C overnight. Peptides were extracted from each gel by adding acetonitrile; the supernatant was collected, and the acetonitrile was removed by using speed-vac (Eppendorf, Hamburg, Germany). The samples were subjected to mass spectrometric analysis using ESI-LC-MS/MS (a micrOTOF-Q instrument, Bruker Daltonics, Bremen, Germany). Each peptide preparation was acidified before injecting into an EASY-nLC system (Bruker Daltonics), and the separation was done at a flow rate 300 nL/min. The eluent was sprayed using a capillary voltage of 22 to 28 kV into a nano-electrospray source of the QToF. The cone was at 100 V; the source temperature was 85 °C, and the microchannel plate detector (MCP) was 2300 V. The MS scan mode covered *m/z* 400–2000. Three most abundant precursors were selected to fragment for 3 s. The MS/MS spectra covered *m/z* 50–1500. For data analysis, the LC-MS/MS data files were searched against Mascot version 2.4.1 (Matrix Science, London, UK) [12], which contained 37,848,116 sequence entries, respectively, was used. Bony vertebrate was set for the taxonomy filter. Missed cleavage was set to 1 with peptide tolerance set to 200 ppm and tandem MS tolerance set to 0.6 Da. Fixed modification was set to carbamidomethyl on cysteine. Variable modifications were set to include methionine oxidation. Only peptides identified above 95% confidence were reported in this study. Each identified peptide was searched against Basic Local Alignment Search Tool (BLAST) [13] for considering isoforms of proteins [14].

### 2.4. Determination of O. hannah Venom Components Cross-Reactive with N. kaouthia Antivenin

*O. hannah* venom was subjected to two-dimensional gel electrophoresis (2DE) [15]. Three aliquots of 75 µg of the *O. hannah* venom were each dissolved in a DeStreak^TM^ rehydration solution that contained pH 3–10 NL IPG buffer (the final volume was 125 µL), and each solution was added into a strip holder of the Ettan IPG Phor Electrofocusing System (Amersham Biosciences). An IPG strip was placed into each strip holder containing the venom (right side down) and allowed to rehydrate at 20 °C for 12 h. Electrophoresis of the IPG strips was performed at 300 V for 30 min, 1000 V for 30 min and 5000 V for 72 min. For the second dimension, the focused IPG strips were equilibrated in a reduction buffer (50 mM Tris-HCl, pH 8.8, 6 M urea, 30% (v/v) glycerol, 2% (w/v) SDS, 0.002% bromophenol blue and 1% (w/v) DTT] at 25 °C for 15 min and in an alkylation buffer (50 mM Tris-HCl, pH 8.8, 6 M urea, 30% (v/v) glycerol, 2% (w/v) SDS, 0.002% bromophenol blue and 2.5% (w/v) iodoacetamide) at 25 °C for 15 min. The SDS-PAGE was carried out in a 15% gel cast in Mini PROTEAN^®^ 3 Cell (Bio-Rad) at 10 mAmp/gel during the first 15 min and 20 mAmp/gel until the tracking dye reached the lower gel edge. One gel was stained by CBB dye; the separated components of the other two gels were transferred to two nitrocellulose membranes (NC) for 2DE-immunoblotting. One NC blot was probed with horse monospecific *N. kaouthia* antivenin (1:100), while another blot was probed with normal horse immunoglobulin (equal protein concentration to 1:100 of antivenin). The *O. hannah* components bound by the horse antibodies were revealed by using goat anti-horse immunoglobulin-alkaline phosphatase (AP) conjugate (Southern Biotech, Birmingham, AL, USA) and 5-bromo-4-chloro-3-indolyl phosphate/nitro blue tetrazolium (BCIP/NBT) substrate (KPL, Gaithersburg, MD, USA). The venom spots on the CBB stained 2DE-gel relevant to the spots that reacted to the horse antivenin on the first 2DE immunoblot membrane, but that did not react to the normal horse immunoglobulin on the second immunoblot membrane were excised out and digested with trypsin. Peptides were subjected to protein identification by ESI-LC-MS/MS using a micrOTOF-Q instrument (Bruker Daltonics, Bremen, German). Each identified peptide was searched against the *O. hannah* nucleotide database.

### 2.5. Median Lethal Dose (LD_50_) of O. hannah Venom in Mice

The LD_50_ of *O. hannah* venom in mice was determined using a previously established method [16,17,18]. Mice (6 mice per group) were injected with varying amounts of *O. hannah* venom in 200 µL of sterile NSS intraperitoneally (i.p.). Alternatively, mice were injected intramuscularly (i.m.) with *O. hannah* venom in 30 µL of sterile NSS in order to simulate the most common route of the snakebites. Control mice received NSS only. The mortality of the mice in all groups was observed, and the experiments were terminated at 48 h post-injection. The LD_50_ of the i.p. and the i.m. injected venom were calculated from two reproducible experiments [19].

### 2.6. Cross-Neutralization of O. hannah Venom by N. kaouthia Antivenin

The cross-species neutralization of the *N. kaouthia* antivenin was performed as described previously [17,18]. Seven groups of 5 mice each (Groups 1–7) were prepared. *O. hannah* venom (1.5 LD_50_) was mixed with the *N. kaouthia* antivenin in 1:5, 1:10, 1:20 or 1:40 (w/w) and kept at 37 °C for 30 min. Individual mice of Groups 1–4 (test groups) were injected i.p. with the venom-antivenin mixtures (500 μL) [16,17,18]. Each mouse of Group 5 received normal horse serum (an equal protein concentration to Group 4) in 500 μL NSS (background neutralization control). Mice of Group 6 received 1.5 LD_50_ *O. hannah* venom in 500 μL NSS (non-neutralization control). Mice of Group 7 received *N. kaouthia* antivenin only (an equal protein concentration to Group 4 in 500 μL NSS) and served as non-envenomed control. The numbers of dead and alive mice at 48 h post-injection were recorded, and the antivenin effective dose (ED_50_) was calculated [19].

Alternatively, six groups of 5 mice each (Groups 1–6) were injected i.m. individually with 1.5 LD_50_ of *O. hannah* venom in 30 µL of NSS (the simulated common route of snakebites). Mice of Group 7 received 30 µL of NSS i.m. Ten minutes later, *N. kaouthia* antivenin (w/w of venom:antibody 1:5, 1:10, 1:20 and 1:40) in 60 µL NSS was injected intravenously (i.v.) (the simulated antivenin treatment route of snakebites in human) into the envenomed mice of Groups 1–4, respectively. Each mouse of Group 5 received normal horse serum (equal protein concentration to the mice of Group 4). Mice of Groups 6 and 7 received 60 µL NSS i.v. alone and the NSS containing *N.* *kaouthia* antivenin (amount of antivenin equal to Group 4), respectively. The numbers of dead and alive mice at 48 h post-venom injection were recorded, and the effective dose (ED_50_) was calculated [19].

### 2.7. Cross-Neutralization of O. hannah Venom by NkLN-HuScFv

Eight mouse groups (5 mice per group) were prepared (Groups 1–8). *O. hannah* venom (1.5 LD_50_) was mixed with NkLN-HuScFv (1:10 or 1:50 w/w in 500 µL of NSS). *O. hannah* venom (1.5 LD_50_) was also mixed with irrelevant HuScFv (HuScFv specific to the NS1 protein of influenza A virus), at amounts equal to 1:10 or 1:50 venom:HuScFv). The mixtures were kept at 37 °C for 30 min. Individual mice of Groups 1 and 3 (test groups) were injected i.p. with venom-NkLN-HuScFv mixtures at 1:10 and 1:50, respectively; mice of Groups 2 and 4 received *O. hannah* venom mixed with influenza virus NS1-specific HuScFv at 1:10 and 1:50, respectively. Mice of Groups 5 and 6 received *O. hannah* venom in buffer (non-neutralization control) and NkLN-HuScFv alone, respectively. Mice of Groups 7 and 8 received 1.5 LD_50_ of *O. hannah* venom mixed with NkLN-HuScFv and irrelevant HuScFv (venom:antibody 1:50) i.p., respectively; ten minutes later, they were given another i.p. dose of NkLN-HuScFv and the irrelevant HuScFv (an antibody amount equal to Groups 3 and 4, respectively). The numbers of dead and alive mice at 48 h post-injection were recorded and the percent of survival of the mice was calculated.

### 2.8. O. hannah Venom Components Cross-Reactive to NkLN-HuScFv

*O. hannah* venom was subjected to 2DE, as described above. The separated components were electroblotted onto two pieces of nitrocellulose membranes (NC) The blotted membranes were kept in a Tris-buffered saline solution containing 3% bovine serum albumin (BSA) and 0.2% gelatin (TBST) at 25 °C for 1 h. After blocking, one blotted membrane was probed with the histidine tagged-NkLN-HuScFv and another was reacted with control histidine-tagged HuScFv (specific to influenza virus matrix protein-1) at 25 °C for 1 h and at 4 °C overnight. Separated *O. hannah* components bound by the HuScFvs were revealed by using the mouse anti-histidine tag antibody, goat anti-mouse immunoglobulin-AP conjugate and BCIP/NBT substrate, respectively. The venom protein spots on the 2DE-gel stained with CBB dye relevant to the spots that appeared on the 2DE-immunoblot membrane probed with NkLN-HuScFv, but that were absent on the membrane probed with the irrelevant HuScFv were excised out and digested with trypsin. Peptides were subjected to protein identification by ESI mass spectrometry using the Ultimate 3000 nano HPLC system (Dionex) coupled to a 4000 Q TRAP mass spectrometer (Applied Biosystems). Tryptic peptides were loaded onto a C18 PepMap100, 3 µm (LC Packings) and separated with a linear gradient of water/acetonitrile/0.1% formic acid (v/v). Spectra were analyzed to identify the proteins of interest using Mascot sequence matching software (Matrix Science) with the Ludwig non-redundant (NR) database.

### 2.9. Computerized Procedure for Determining the Interactions between NkLN-HuScFv and Long Neurotoxins of N. kaouthia and O. hannah

Interactions between NkLN-HuScFv with *N. kaouthia* and *O. hannah* long neurotoxins were elucidated by using computerized simulation. The amino acid sequences of the venom proteins were obtained from the UniProt Protein Knowledgebase [20]. The venom protein sequences were used for searching experimental three-dimensional (3D) structures of the venom components by means of the Protein Model Portal [21] from the Research Collaboratory for Structural Bioinformatics (RCSB) protein data bank [22]. Only the 3D structure of *N. kaouthia* long neurotoxin was available. Thus, the amino acid sequence of the *O. hannah* long neurotoxin, as well as the NkLN-HuScFv sequence were subjected to homology modeling [23,24] by the Iterative Threading Assemble Refinement (I-TASSER) server service [25]. The predicted models derived from I-TASSER were subsequently refined by using two server services, *i.e.*, the high-resolution protein structure refinement, ModRefiner [26,27], and the fragment-guided molecular dynamics (FG-MD) simulation [28,29]. The local geometric and physical qualities of the predicted 3D structures were improved to make them come closer to their native state. The refined models were docked according to a fast Fourier transform (FFT)-based program, *i.e.*, PIPER. The antibody-venom component dockings were performed by using the antibody mode available on the automated ClusPro 2.0 protein-protein docking server [30,31,32,33,34]. The largest cluster size, which indicated a region of local minimum energy and a near-native state protein conformation, was chosen for each docking. The protein structure models and the molecular interactions were built and visualized by using PyMOL software (The PyMOL Molecular Graphics System, Version 1.3r1 edu, Schrodinger, LLC, NY, USA).

## 3. Results

### 3.1. O. hannah Venom Proteome

**Figure 1:**
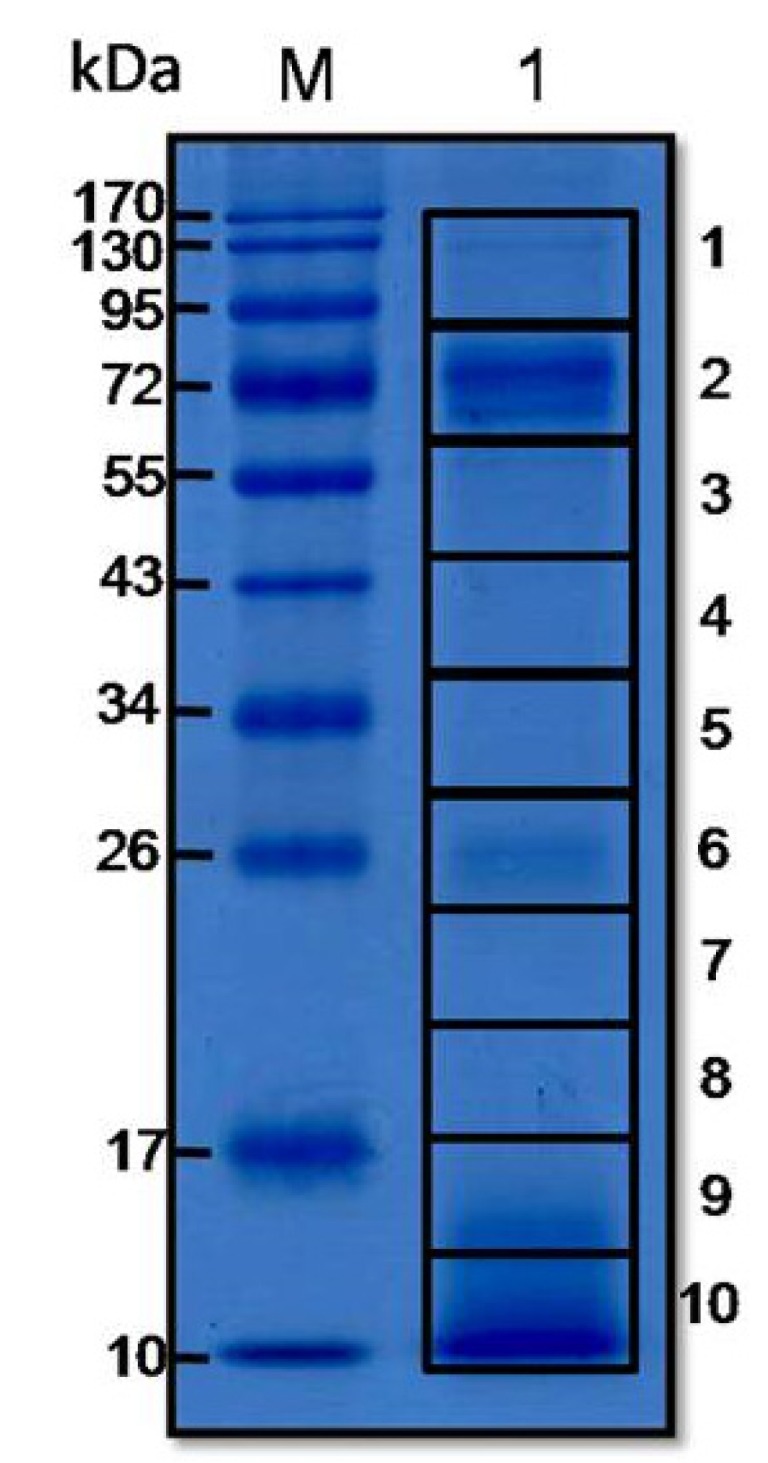
SDS-PAGE separated pattern of *O. hannah* holovenom. M, protein molecular weight standard. Lane 1, SDS-PAGE separated components of the venom stained with Coomassie Brilliant Blue R-250 (CBB) dye. Numbers on the right are the gel pieces containing the *O. hannah* proteins that were subjected to protein identification by LC-MS/MS. Numbers at the left are protein molecular masses (kDa).

The proteins of the *O. hannah* venom ranged in masses from <10 to 130 kDa (Figure 1). The trypsin digested peptides of proteins in each gel piece of the Figure 1 were subjected to ESI-LC-MS/MS analysis and database search. The *O. hannah* venom peptides matched with the peptides of the proteins of the database are shown in Table 1. They could be classified into 14 different protein types/families (Table A1), *i.e.*, three finger toxin, phospholipase, cysteine-rich secretory protein (CRiSP), cobra venom factor/complement C3, muscarinic toxin, L-amino acid oxidase, hypothetical protein, low cysteine protein, phosphodiesterase, protease, Vespryn toxin, Kunitz, growth factor activator and others (coagulation factor, endonuclease, 5’-nucleotidase).

### 3.2. O. hannah Venom Components Cross-Reactive to N. kaouthia Antivenin

**Figure 2:**
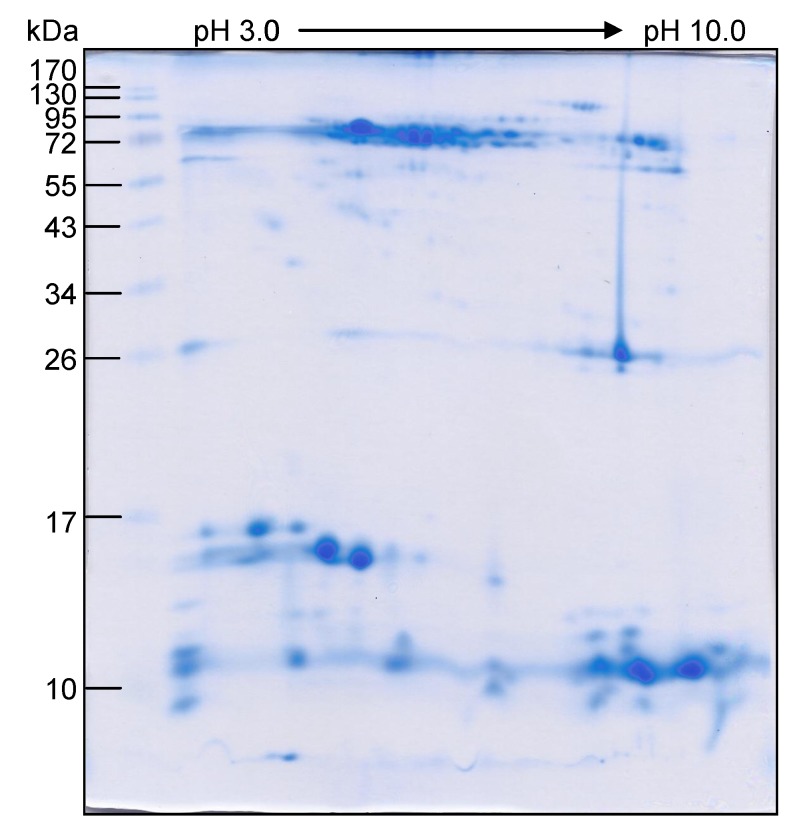
Pattern of *O. hannah* holovenom proteins after two-dimensional gel electrophoresis (2DE) and CBB staining. Numbers at the left are protein masses in kDa.

**Figure 3:**
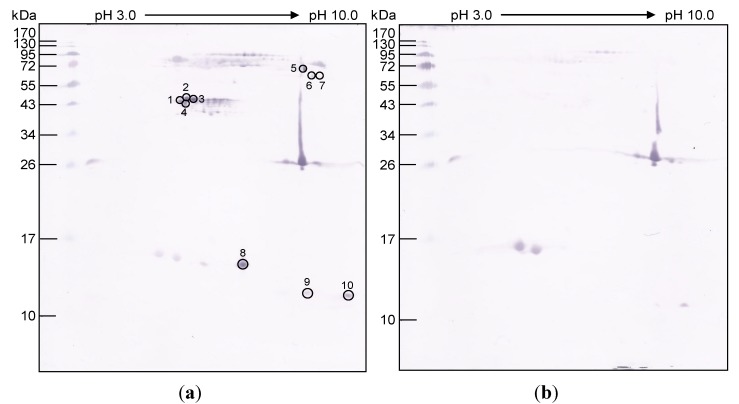
Patterns of *O. hannah* venom after 2DE at non-linear pH 3.0–10.0 and probed with (**a**) horse-derived *N. kaouthia* antivenin and (**b**) normal horse serum. Encircled spots and numbers are *O. hannah* proteins that reacted with the antivenin and did not react with the normal horse serum.

The majority of the venom proteins after 2DE and CBB staining were located in two areas: the ~10–17 and ~26–130 kDa regions (Figure 2). Ten protein spots reacted to the *N. kaouthia* antivenin, but did not react to the normal horse immunoglobulin (Figure 3). The *O. hannah* venom peptides generated from the protein spots that matched with the protein sequences of the database are shown in Table 2. They could be classified into six functionally different proteins (Table A2) including: cobra venom factor/complement C3, *i.e.*, *O. hannah* venom factor precursor protein (Spots 1 and 3); cardiotoxin-like protein (three finger toxin) (Spot 2); cysteine-rich secretory protein (CRiSP), ophanin/opharin/opharin precursor (Spots 5 and 6); weak neurotoxin precursor (three finger toxin) (Spots 7 and 10); long chain neurotoxin (three finger toxin) (Spots 8–10); short chain α-neurotoxin (three finger toxin) (Spots 9 and 10). No protein in the database matched the peptides of spot 4.

**Table 1:** LC-MS/MS Mascot results of in-gel tryptic digestions of *O. hannah* venom searching against the NCBInr database.

Gel piece No.	Protein No.	Accession No.	Protein	Protein score	Mass	*m/z*	z	Peptide score	Sequence
1	1	gi|391359389	Zinc	911	69,003	650.38	2	43.19	NNLLHFSIWR
			metalloproteinase-			799.97	2	64.70	TNNVIIPCKPTDVK
			disintegrin-like			821.39	2	102.49	LYCTGGTENPSEGEK
			ohanin			861.43	2	96.53	ASYSEIEDIGMVDHR
						957.01	2	136.20	AMCDVLQSVGIVQDYSK
						1,214.54	2	126.32	NECDLPEFCIGQSAECPMDR
						810.39	3	78.10	LYCTGGTENPSEGEKISSDPCK
						1,220.67	2	122.89	NDNAQLLTGVDLNGYTLGSAYLK
						1,222.53	2	83.89	NECDLPEFCIGQSAECPMDR
						860.11	3	74.59	SPYLVGAAMAHEIGHNLGMEHDTK
						947.44	3	65.64	NECDLPEFCIGQSAECPMDRFHK
1	2	gi|347595788	L-amino-acid oxidase	537	55,941	690.87	2	62.68	QTDENAWYLIK
						749.50	2	69.76	KIVIVGAGISGLTAAK
						762.92	2	66.38	EAGHEVVILEASDR
						776.45	2	64.94	IILVCTDKFWEK
						1,056.01	2	117.64	MSANNPENFGYQLNPNER
						780.07	3	63.43	IYFAGEYTAHPHGWIETSMK
						805.42	3	91.79	SASQLFDETLDKVTDDCTLQK
1	3	gi|22654267	Acidic phospholipase	510	16,432	792.41	2	101.70	LPACSSIMDSPYVK
			A2			906.40	2	86.21	CCQVHDNCYTQAQK
						915.89	2	94.20	ADNDECAAFICNCDR
						794.71	3	129.81	YADYGCYCGAGGSGTPVDKLDR
						872.10	3	97.84	VAAHCFAASPYNNNNYNIDTTTR
1	4	gi|126035663	Complement-	235	184,237	955.61	2	124.43	LILNTPLDTQSLLITVR
			depleting factor			999.02	2	68.09	TDTEEQILVEAHGDNTPK
			(*O. hannah*)			1,065.65	2	42.31	AVPFVIVPLQQGLHDIEVR
1	5	gi|82193162	Long neurotoxin OH-37	183	9846	670.36	2	91.89	KLSFGCAATCPK
						1,164.04	2	91.17	VNPGIDIECCSTDNCNPHPK
1	6	gi|338855302	Phosphodiesterase 1	160	96,311	771.08	3	95.44	NPAWWGGQPIWHTATYQGLK
						1,216.13	2	65.03	NEVTSFENIEVYNLMCDLLK
1	7	gi|82193155	Short neurotoxin OH-35	126	9558	1,239.13	2	126.42	QYTIFGVTPEICADGQNLCYK
1	8	gi|565306229	Insulin-like growth factor I (*O. hannah*)	115	12,329	936.45	2	115.04	GIVEECCFQSCDLVR
1	9	gi|565293365	Hypothetical protein L345_17517, partial (*O. hannah*)	65	25,866	777.87	2	64.55	GIDSSHWNSYCTK
1	10	gi|391359387	Zinc metalloproteinase-disintegrin-like mikarin	63	18,426	796.44	2	62.61	TNTPEQDRYLQVK
2	11	gi|544604740	*Ophiophagus* venom	534	183,812	606.86	2	43.11	AVYVLNDKYK
			factor			8,867.00	2	48.71	YVLPSFEVHLQPSEK
						905.04	2	58.82	IKLEGDPGAQVGLVAVDK
						955.61	2	118.78	LILNTPLDTQSLLITVR
						999.02	2	104.68	TDTEEQILVEAHGDNTPK
						1,010.07	2	94.74	YLYGEEVEGVAFVLFGVK
						700.05	3	67.01	VPVVSEAIHSEGTTLSDGTAK
						810.39	3	43.35	LYCTGGTENPSEGEKISSDPCK
						749.51	2	96.89	KVVIIGAGISGLTAAK
3	12	gi|565315338	5~-nucleotidase, partial (*O. hannah*)	53	28,027	958.05	2	53.17	VLLPSFLAAGGDGYYMLK
4	13	gi|387935404	Alpha- and beta-	510	28,637	573.30	2	95.85	GDSGGPLICNR
			fibrinogenase OHS1			812.40	2	105.35	IIGGFECNEYEHR
						818.40	2	77.40	DSCKGDSGGPLICNR
						881.50	2	102.54	VMGWGLLTSPEVTFPK
						1,325.22	2	128.74	EIQGIVSWGGFPCAQLLEPGVY TK
4	14	gi|565314693	Hypothetical protein	266	24,222	573.30	2	95.85	GDSGGPLICNR
			L345_07470, partial			818.40	2	77.40	DSCKGDSGGPLICNR
			(*O. hannah*)			1,317.72	2	92.89	QIQGVVSWGGFPCAQLLEPGVYT K
4	15	gi|565308117	hypothetical protein	206	115,185	650.38	2	49.92	NNLLHFSIWR
			L345_12124			818.40	2	77.40	DSCKGDSGGPLICNR
			(*O. hannah*)			871.39	2	82.22	NGHSCQNNQGYCFR
						957.00	2	74.28	AMCDVLQSVGIVQDYSK
4	16	gi|54035743	Cobra venom factor	64	184,401	841.91	2	63.74	VYSYYNLDEKCTK
5	17	gi|565307033	Hepatocyte growth	174	10,782	981.57	2	104.44	YSNVVQEALIPIIPDYK
			factor activator,			1,161.59	2	69.52	FIQPICLPEASMSFPDYYK
			partial (*O. hannah*)			937.51	2	69.87	GILDENQWESGLFLPR
5	18	gi|118151738	Metalloproteinase precursor (*Demansia vestigiata*)	62	68,267	649.30	2	61.92	SAECPTDSFQR
5	19	gi|18000318	Cysteine-rich venom protein (*Hydrophis hardwickii*)	58	20,109	888.95	2	57.69	YLYVCQYCPAGNIR
5	20	gi|182705250	Zinc metalloproteinase/ disintegrin	53	71,170	957.00	2	53.19	GMCDPKLSVGLVQDYSK
6	22	gi|565292399	Endonuclease domain-	234	18,064	848.51	2	75.99	SSTFTLTNIVPQFIK
			containing 1 protein			1,055.56	2	66.13	GCQQTFAVVGAVPGDTYIAR
			(*O. hannah*)			1,162.10	2	94.19	ALQDSQAVLEDYKNLADCNR
						888.95	2	74.76	YLYVCQYCPAGNIR
6	23	gi|1584763	Phospholipase A2	137	13,447	915.89	2	58.19	ADNDECAAFICNCDR
						794.71	3	79.24	YADYGCYCGAGGSGTPVDKLDR
6	24	gi|565304281	Endonuclease domain-containing 1 protein, partial (*O. hannah*)	126	51,035	628.36	2	49.98	FATLYDKQNR
6	26	gi|565303552	Phospholipase B-like 1, partial (*O. hannah*)	71	58,242	869.47	2	73.13	DLHYATVYWLEAEK
7	27	gi|225547744	Opharin precursor	317	26,288	640.78	2	43.67	CPASCFCHNK
			(*O. hannah*)			788.39	2	78.40	YKDDFSNCQSLAK
						888.95	2	77.95	YLYVCQYCPAGNIR
						1,092.02	2	68.72	FSCGENLFMSSQPYAWSR
						1,212.51	2	48.66	SGPPCGDCPSACDNGLCTNPCK
8	29	gi|124020977	PLA_2_ Hs-1 precursor (*Hoplocephalus stephensii*)	61		915.88	2	60.84	ADNDECKAFICNCDR
9	30	gi|48428841	Cysteine-rich venom protein	123	26,229	705.39	2	56.89	VIQSWYDENKK
			natrin-2			925.46	2	66.57	NMLQMEWNSNAAQNAK
9	31	gi|82193154	Cardiotoxin CTX15	104	9,346	545.84	2	60.05	LPSKYDVIR
						666.40	2	43.75	CLNTPLPLIYK
						738.43	2	42.00	FLEQQNQVLQTK
9	32	gi|32363235	Thai cobrin	80	12,087	757.41	3	80.32	ADVTFDSNTAFESLVVSPDKK
9	33	gi|26006829	Acidic phospholipase	61	15,890	847.13	3	61.15	VAAICFAGAPYNKENINIDTTTR
			A2-2			792.41	2	105.12	LPACSSIMDSPYVK
						906.41	2	77.19	CCQVHDNCYTQAQK
						915.89	2	94.44	ADNDECAAFICNCDR
						794.71	3	147.85	YADYGCYCGAGGSGTPVDKLDR
						1,210.54	2	92.09	TVTCKADNDECAAFICNCDR
						872.10	3	101.16	VAAHCFAASPYNNNNYNIDTTTR
						925.45	3	109.09	VAAHCFAASPYNNNNYNIDTTTRC
10	34	gi|82199673	Cytotoxin-27	251	9,305	799.38	2	67.56	SSADVEVLCCDTNK
			(ß Cytotoxin)			1,102.61	2	115.40	CLNTPLPLIYTTCPIGQDK
						1,138.12	2	68.32	KCLNTPLPLIYTTCPIGQDK
10	35	gi|82175774	Short neurotoxin	247	8,886	560.25	2	47.13	YSVCCSTDK
			OH-46			761.34	2	62.70	YSVCCSTDKCNK
						929.47	3	137.57	TTMFFPNHPVLLMGCTYNCPTER
10	36	gi|123916245	Cardiotoxin CTX21	242	9,249	978.48	2	58.44	SSADVVVVCCDTNKCNK
						1,102.61	2	115.4	CLNTPLPLIYTTCPIGQDK
						1,138.12	2	68.32	KCLNTPLPLIYTTCPIGQDK
10	37	gi|116242842	Ohanin	177	21,161	749.40	2	88.66	FSSSPCVLGSPGFR
						1,135.61	2	88.46	ADVTFDSNTAFESLVVSPDKK
						1,239.13	2	140.55	QYTIFGVTPEICADGQNLCYK
						666.40	2	59.72	CLNTPLPLIYK
						799.38	2	67.56	SSADVEVLCCDTNK
						1,022.51	2	64.29	VSETIEICPDGQNFCFK
10	40	gi|239977312	Protease inhibitor TCI	105	8,976	998.00	2	104.67	AYIPSFYYNPDASACQK
10	41	gi|123913366	Weak neurotoxin WNTX-34	98	9,808	826.35	2	97.90	EIVQCCSTDECNH
10	42	gi|82193157	Long neurotoxin OH-17	88	8,032	893.90	2	88.18	CCSTDNCNPFTPWK
10	43	gi|82193161	Long neurotoxin OH-55	82	10,203	1,013.59	2	81.91	VNLGCAATCPIVKPGVEIK
10	44	gi|128951	Long neurotoxin-4	75	8,009	941.47	2	75.32	IISEACPPGQDLCYMK
10	45	gi|83305935	Neurotoxin-like protein 1	74	7,037	625.83	2	74.01	KDEVIQCCAK
10	46	gi|182705233	Weak toxin DE-1	74	9,297	650.87	2	73.67	VNPPISIICCK
10	47	gi|239938646	Venom chymotrypsin inhibitor	52	9,130	830.42	2	52.00	FCELPPEPGLCNAR
10	48	gi|123907650	Cardiotoxin CTX-23	146	9,290	459.74	2	33.85	TCPIGQDK
						666.40	2	58.07	CLNTPLPLIYK
						978.48	2	54.36	SSADVVVVCCDTNKCNK
10	49	gi|123913365	Muscarinic toxin-38	48	9,678	756.38	2	48.13	TCPDGQNLCYKR
10	50	gi|128944	Long neurotoxin-2	70		893.90	2	69.94	CCSTDNCNPFPTWK

**Table 2:** *O. hannah* venom components cross-reactive with horse-derived *N. kaouthia* antivenin.

Gel piece No.	Accession No.	Protein	Protein score	Mass	*m/z*	z	Peptide score	Sequence
1	gi|544604740	*O. hannah* venom factor	54	183,812	428.26	2	29.96	VVPEGVQK
					710.74	3	26.31	AVPFVIVPLQQGLHDIEVR
2	gi|38049474	Cardiotoxin-like protein	187	9,818	481.73	2	38.14	GCIDICPK
		(*O. hannah*)		9,818	545.82	3	27.06	LPSKYDVIR
				9,818	799.36	2	65.14	SSADVEVLCCDTNK
				9,818	735.39	3	64.75	CLNTPLPLIYTTCPIGQDK
3	gi|387165368	OVF precursor protein (*O. hannah*)	52	185,408	482.26	3	27.04	HFEVGFIQPGSVK
			52	185,408	710.74	3	25.54	AVPFVIVPLQQGLHDIEVR
4	No matched protein in the database							
5	gi|28972961	Ophanin (*O. hannah*)	82	27,764	567.79	2	29.55	SVSPTASNMLK
				27,764	640.78	2	54.73	QSSCQDEWIK
6	gi|225547744	Opharin precursor	46	27,200	418.73	2	26.72	GSIATPYK
		(*O. hannah*)		27,200	525.91	2	20.06	YKDDFSNCQSLAK
7	gi|82570105	Weak neurotoxin precursor	53	10,492	484.23	2	28.00	NGENVCFK
		(*O. hannah*)		10,492	551.21	3	25.30	EIVQCCSTDECNH
8	gi|82570073	Long chain neurotoxin precursor (*O. hannah*)	25	11,155	644.28	2	24.64	TWCDVFCGSR
9	gi|82570073	Long chain neurotoxin	60	11,155	644.20	2	27.10	TWCDVFCGSR
		precursor (*O. hannah*)			956.40	2	32.50	IISETCPPGQDLCYMK
	gi|51105381	Long chain neurotoxin	123	8,602	603.20	2	49.40	IDLGCAATCPK
		precursor (*O. hannah*)			659.20	2	50.12	SWCDVFCTSR
					837.30	2	23.54	SETCPDGENICYTK
	gi|51105369	Long chain neurotoxin	60	10,477	606.20	2	27.95	LSFGCAATCPK
		precursor (*O. hannah*)			642.70	2	32.33	SWCDAWCGSR
9	gi|51105375	Long chain neurotoxin	38	10,538	595.70	2	37.91	VNLGCAATCPK
		precursor (*O. hannah*)						
	gi|82570079	Long chain neurotoxin	74	10,944	491.20	2	41.32	CYVTPDVK
		precursor (*O. hannah*)			837.30	2	32.65	SETCPDGENICYTK
	gi|128944	Long neurotoxin-2	72	8,602	603.20	2	43.12	IDLGCAATCPK
		(Neurotoxin B)			937.00	3	28.64	TKCYVTPDATSQTCPDGQDICYTK
	gi|51105373	Long chain neurotoxin	77	10,830	491.20	2	31.89	CYVTPDVK
		precursor (*O. hannah*)			676.00	3	45.21	VNLGCAATCPIVKPGVEIK
	gi|51105391	Short chain alpha neurotoxin precursor (*O. hannah*)	36	10,071	826.30	3	35.67	QYTIFGVTPEICADGQNLCYK
10	gi|51105397	Short chain alpha	114	9,859	481.70	2	29.87	GCIDICPK
		neurotoxin precursor			666.30	2	36.34	CLNTPLPLIYK
		(*O. hannah*)			667.20	3	47.91	SSADVEVLCCDTNKCNK
	gi|82570105	Weak neurotoxin	46	10,492	826.20	2	46.23	EIVQCCSTDECNH
		precursor (*O. hannah*)			799.30	2	41.31	SSADVEVLCCDTNK
					667.20	3	27.16	SSADVEVLCCDTNKCNK
	gi|82570079	Long chain neurotoxin	146	10,944	491.20	2	38.11	CYVTPDVK
		precursor (*O. hannah*)			837.30	2	36.70	SETCPDGENICYTK
					596.20	3	27.89	CCSTDNCNPFTPWK
					648.20	3	43.67	CCSTDNCNPFTPWKR
	gi|128944	Long neurotoxin 2	167	8,602	603.20	2	27.85	IDLGCAATCPK
		(Neurotoxin B)			596.20	3	33.17	CCSTDNCNPFPTWK

**Table 3:** Efficacy of HuScFv specific to *N. kaouthia* long neurotoxin (NkLN-HuScFv) in the cross-neutralization of *O. hannah* venom.

Group of mice	Treatment (injected i.p. with)	Ratio of venom : HuScFv (w/w)	Mean ± SD of mouse dead time ( min)	% Survival
1	Venom-NkLN-HuScFv mixture	1:10	111 ± 16.5 ^a^	0
2	Venom-irrelevant HuScFv mixture	1:10	88 ± 7.2 ^b^	0
3	Venom-NkLN-HuScFv mixture	1:50	155 ± 58.7 ^a^	0
4	Venom-irrelevant HuScFv mixture	1:50	93 ± 6.8 ^b^	0
5	Venom in buffer	NA	78 ± 7.3 ^b^	0
6	NKLN-HuScFv alone	NA	All mice survived at the end of experiments	100
7	Venom-NkLN-HuScFv mixture and followed 10 min later with NkLN-HuScFv alone	1:50	220 ^a^	80
8	Venom-irrelevant HuScFv mixture and followed 10 min later with irrelevant HuScFv alone	1:50	95 ± 24.4 ^b^	0

Notes: NA, not available. Entries with different superscripts (a *versus* b) were different significantly at *p* < 0.05 (*t*-test). Irrelevant HuScFv were specific to the influenza A virus M1 protein.

### 3.3. Median Lethal Dose (LD_50_) of O. hannah Venom and the Venom Cross-Neutralization by the Monospecific N. kaouthia Antivenin

The LD_50_ of the *O. hannah* venom injected i.p. and i.m. into mice were 1.1 and 0.59 µg/g of body weight, respectively. All mice that received 1.5 LD_50_ of the *O. hannah* venom either i.p. or i.m. died within 48 h post-injection. Envenomed mice that received only normal horse serum were all dead within 48 h post-injection also, while mice that received *N. kaouthia* antivenin alone survived with no detectable adverse effect. The ED_50_ of the *N. kaouthia* antivenin mixed with 1.5 LD_50_ *O. hannah* venom and injected i.p. to mice was 590 µg per mouse (19.6 µg/g body weight). When used to treat the mice that received 1.5 LD_50_ of *O. hannah* venom i.m., the i.v. antivenin ED_50_ was 404 μg in 60 µL of NSS per mouse or 13.5 μg/g body weight.

### 3.4. Cross-Neutralization of O. hannah Venom by HuScFv Specific to N. kaouthia Neurotoxin

Mice injected with a mixture of *O. hannah* venom (1.5 LD_50_) and NkLN-HuScFv at 1:10 and 1:50 (w/w) (Groups 1 and 2, respectively) had significantly longer death times (111 ± 16.5 and 155 ± 58.7 min, respectively) compared with the mice injected with the venom alone (78 ± 7.3 min) (*p* = 0.00008 and 0.0004, respectively) (Table 3). The means ± SD of the death times of NkLN-HuScFv treated mice at the venom:HuScFv ratios of 1:10 and 1:50 were also longer than the irrelevant HuScFv neutralization controls (*p* = 0.01 and 0.04, respectively). Eighty percent of mice that were injected with *O. hannah* venom (1.5 LD_50_) mixed with NkLN-HuScFv (venom:HuScFv ratio 1:50 w/w) i.p. and 10 min later received another therapeutic dose of NkLN-HuScFv survived, while all mice that received 1.5 LD_50_ of the *O. hannah* venom alone or mixed with irrelevant HuScFv i.p. and followed 10 min later by NSS and the irrelevant HuScFv i.p., respectively, died within 48 h post-injection (Table 3).

### 3.5. O. hannah Venom Components Cross-Reactive to NkLN-HuScFv

**Figure 4:**
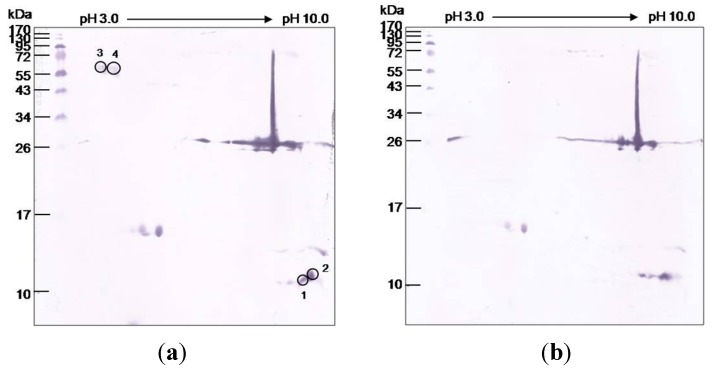
*O. hannah* venom proteins after 2DE and probed with (**a**) NkLN-HuScFv and (**b**) irrelevant HuScFv (control). Encircled spots are the *O. hannah* proteins that reacted with NkLN*-*HuScFv on the 2DE immunoblot membrane, but did not react with the irrelevant HuScFv (specific to the M1 protein of influenza A virus). Proteins in spots 1 and 2 were identified as *O. hannah* three finger toxins, *i.e.*, short and long neurotoxins. No proteins in the database matched with the proteins of spots 3 and 4.

There were four protein spots (1–4) of the 2DE-*O. hannah* venom that were bound by the NkLN-HuScFv (Figure 4a), but that were not bound by the irrelevant HuScFv (Figure 4b). The proteins in the spots 1 and 2 were identified as three finger toxins, *i.e.*, short and long chain neurotoxins, while no protein in the database matched with the proteins of the spots 3 and 4.

### 3.6. Computerized Models of Interactions between N. kaouthia and O. hannah Long Neurotoxins and NkLN-HuScFv

**Figure 5:**
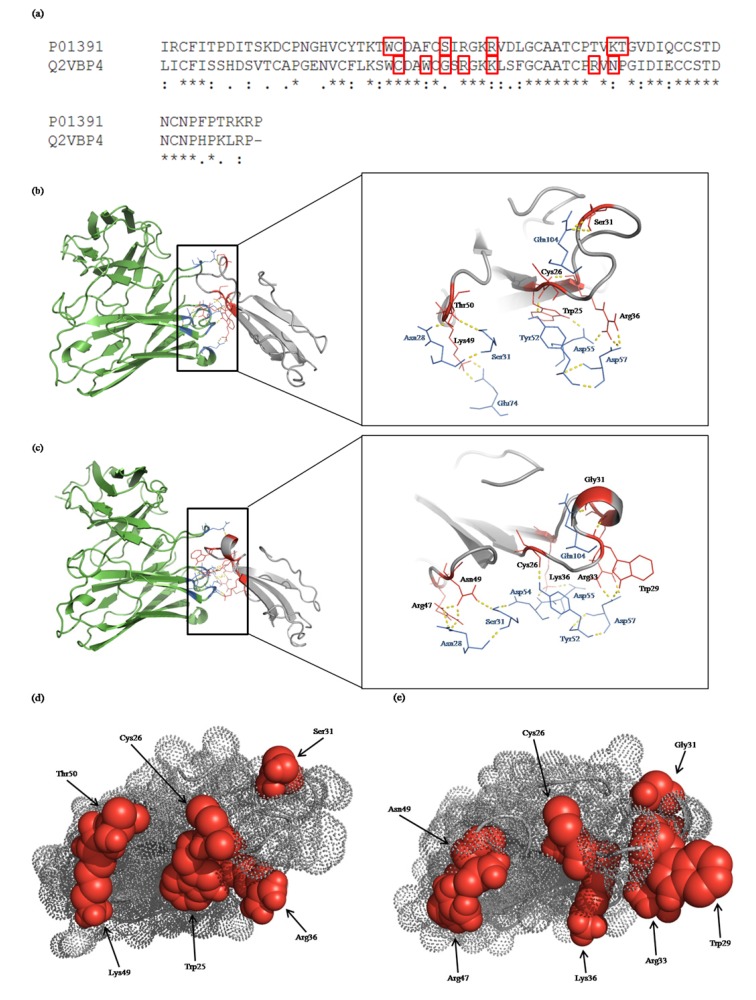
(**a**) Alignments of amino acid sequences of *N. kaouthia* and *O. hannah* neurotoxins. The residues in the epitopes recognized by NkLN-HuScFv are contained in the red boxes. (**b**,**c**) Illustrations of the computerized molecular dockings between NkLN-HuScFv (green ribbon) with the long neurotoxins (gray ribbons) of *N. kaouthia* and *O. hannah,* respectively. (**d**,**e**) Space-filling illustrations of the computerized models of the neurotoxins of *N. kaouthia* and *O. hannah,* respectively. NkLN-HuScFv interactive amino acids of the toxins are shown in red ball-like structures. NkLN-HuScFv interacted with amino acids W25, C26, S31 and R36 in loop 2 and K49 and T50 in loop 3 of the *N. kaouthia* long neurotoxin and with the C26, W29, G31, R33 and K36 in loop 2 and R47 and N49 in loop 3 of the *O. hannah* long neurotoxin.

The crystal structure of *N. kaouthia* long neurotoxin (CBTX) was found in the database (PDB ID: 1CTX), while the 3D structures of the *O. hannah* long neurotoxin (OH-LNTX28) and NkLN-HuScFv had to be modeled by the I-TASSER service. The modeled scores of the OH-LNTX28 and NkLN-HuScFv were 1.16 and 1.14, while the expected template-modeling (TM) scores were both 0.87 ± 0.07 and the expected similarity (RMSD) scores were 1.2 ± 1.2 and 3.5 ± 2.4 Å, respectively, which indicated the appropriateness of the modeled structures. The details of the interactions between *N. kaouthia* long neurotoxin (CBTX) and *O. hannah* long neurotoxin (OH-LNTX28) with NkLN-HuScFv are shown in Table 4 and Figure 5. The alignments of the amino acid sequences of *N. kaouthia* and *O. hannah* neurotoxins and the residues of both toxins recognized by NkLN-HuScFv are shown in Figure 5a. The largest clusters obtained from ClusPro 2.0 were 274 and 215 members, respectively. The lowest energies of the representative models of the molecular dockings were −262.9 and −281.2 kcal/mol, respectively. According to the docking outputs, NKLN-HuScFv used the amino acids D55, Y52, Q104, D55/D57, E74 and N28/S31 in the VH domain to interact with six amino acids of the CBTX, including W25, C26, S31 and R36 in loop 2 and K49 and T50 in loop 3 of *N. kaouthia* long neurotoxin, respectively (Table 4 and Figure 5b,d). The antibody used the VH residues, including Y52, D57, Q104, D57, D54/D55, N28 and S31 of CDRs1-3, to dock on *O. hannah* mature long neurotoxin (Q2VBP4) residues C26, W29, G31, R33 and K36 in loop 2 and R47 and N49 in loop 3 of the *O. hannah* long neurotoxin, respectively (Table 4 and Figure 5c,e).

**Table 4:** Residues of *N. kaouthia* and *O. hannah* long neurotoxins that interacted with NkLN-HuScFv.

Neurotoxin	NkLN-HuScFv	
	Residue(s)	Domain/Subdomain
**CBTX (P01391)**		
W25	D55	VH/CDR2
C26	Y52	VH/CDR2
S31	Q104	VH/CDR3
R36	D55, D57	VH /CDR2
K49	E74	VH/FR3
T50	N28, S31	VH/CDR1
**OH-LNTX (Q2VBP4)**		
C26	Y52	VH/CDR2
W29	D57	VH/CDR2
G31	Q104	VH/CDR3
R33	D57	VH/CDR2
K36	D54, D55	VH/CDR2
R47	N28	VH/CDR1
N49	S31	VH/CDR1

## 4. Discussion

In this study, 1DE gel-based proteomic analysis was used to reveal the *O. hannah* venom proteome. The venom proteins ranged in sizes from <10 to 170 kDa, which conformed to the previous data [35]. The *O. hannah* venom showed two major protein bands at 10–17 and 72 kDa, which, more or less, similar to the Malaysian *O. hannah* venom that had predominant bands at 15–20 and 100 kDa [36]. The differences observed in different studies should be due to the different geographical areas of the cobras, as well as the technical details [37].

*O. hannah* venom components have been studied previously [38,39,40]. By using 2DE and MALDI-ToF mass spectrometry [38], 12 venom proteins were reported, *i.e.*, weak toxin DE-1, cysteine-rich secretory protein, metallothionein-3, metallothionein, hypothetical proteins, putative DNA invertase, ribosomal protein S17P, small heat stress protein class CIII, XYLDLEGF operon transcriptional activator-II, Phe-Met-Arg-Phe amide neuropeptide precursor, cytochrome c-553 precursor and protein similar to the microtubule-associated RP/EB family. Among these proteins, only the weak toxin, DE-1 and cysteine-rich secretory protein were toxic. Seventeen different protein families in the venom gland of Indonesian king cobra (*Ophiophagus hannah*) were predicted by using a draft genome and deep transcriptome sequencing [39], *i.e.*, three finger toxin, phospholipase-A2 (PLA_2_), acetylcholine esterase, metalloproteinases, cysteine-rich secretory protein, cobra venom factor (CVF), hyaluronidase (HYA), Kunitz (serine protease inhibitor), lectin, nerve growth factor (NGF), natriuretic peptide (NP), peptidase, phospholipase-B (PLB), vascular endothelial growth factor (VEGF), ohanin, waprin (protease inhibitor) and various other proteins. Recently, pooled samples of king cobra venom from Indonesia, Malaysia, Thailand and two provinces of China were found to contain eight novel PLA_2_s. Three finger toxins, *i.e.*, OH-55 (long neurotoxin) and OH-27 (beta cardiotoxin), and the Kunitz inhibitor (OH-TCI) were common in all of the five *O. hannah* venoms. Southeast Asian *O. hannah* venoms contained higher metalloproteinase, acetylcholine esterase and alkaline phosphatase than the Chinese venoms [41]. In this study, *O. hannah* venom proteome were revealed by 1 DE and ESI-LC-MS/MS and found that peptides of the venom matched with 14 different protein types/families of the database. Among them, long and short neurotoxins, weak neurotoxin, muscarinic toxin, phospholipase-A_2_ cardiotoxins, phospholipase-B, zinc metalloproteinases, ohanin, serine protease inhibitor, cobra venom factor (CVF) and cysteine-rich secretory protein (ophanin) proteins were found, which verified the transcriptomic data reported previously [39].

The majority of the venom proteins located at ~10–17 and ~26–130 kDa after 2DE and CBB staining, which reproduced the 1DE data and was relatively similar to the 2DE pattern of *O. hannah* venom reported previously [36,38]. *N. kaouthia* antivenin reacted with ten *O. hannah* proteins, all of which were toxic. *N. kaouthia* and *O. hannah* were both elapid snakes and their venoms shared common components [38,41]. The two snake species also have the same geographical and diet predilection [42]. Therefore, the *in vivo* cross-neutralization of monospecific *N. kaouthia* antivenin against the *O. hannah* venom could be expected.

Cross-neutralization of horse-derived monospecific *N. kaouthia* antivenin of QSMI to *O. hannah* venom was as expected. The effective dose fifty (ED_50_) of the *N. kaouthia* antivenin when mixed with the *O. hannah* venom (1.5 LD_50_) and injected i.p. into the mice was 19.6 µg/g body weight, while the ED_50_ of the antivenin when used to treat the envenomized mice that received the 1.5 LD_50_ of *O. hannah* venom i.m. followed by the antivenin intravenously was 13.5 μg/g body weight. Leong *et al.* [43] reported that the ED_50_ of the QSMI horse-derived *N. kaouthia* antivenom when incubated at 37 °C for 30 min with 2.5 LD_50_ of *O. hannah* venom and injecting the mixtures into the caudal veins of mice was 2.80–3.35 (average 3.07) mg/mL. Because the amount of the antivenin injected into individual mice in the study [43] was not mentioned and also due to the different LD_50_ used, the ED_50_ of the antivenins in the previous and the present studies could not be compared.

The gene sequence coding for HuScFv of clone 8/22/3 [10] that neutralized *N. kaouthia* neurotoxin and that rescued 100% of the *N. kaouthia* envenomized mice from lethality was subcloned from pCANTAB5E phagemid vector into pET23b^+^ plasmid vector in order to improve the HuScFv expression yield. The mice that received intraperitoneally the mixtures of *O. hannah* venom and NkLN-HuScFv at 1:10 and 1:50 showed significantly longer means ± SD of death times than the non-NkLN-HuScFv controls (*p* < 0.05). Nevertheless, when another therapeutic dose of the NKLN-HuScFv was given to the mice that received the mixture of 1:50 venom:NkLN-HuScFv, 80% of the mice survived at the end of the experiments, indicating that the specific HuScFv could rescue the mice from *O. hannah* envenomation in a dose-dependent manner. By the 2DE immunoblotting, NkLN-HuScFv bound to the short and long chain neurotoxins of the *O. hannah* venom. Thus, the NkLN-HuScFv should mediate the paraspecific protection of the *O. hannah* envenomed mice by neutralizing these lethal venom components.

The 2DE proteomics and the 2DE western blot analysis used for revealing the components of the *O. hannah* venom and the components recognized by the antibody, respectively, have some limitations. The 2DE method may not recognize components present in minute amounts in the holovenom [41]. The 2DE immunoblotting provided a qualitative assessment and did not provide the degree of immunoreactivity (avidity/affinity) of each antigen-antibody interaction. Besides, the antibodies could recognize only the denatured venom components in the assay and, thus, could not reveal the binding of conformational epitopes present in the native proteins.

According to the molecular docking results, NkLN-HuScFv used all three CDRs, as well as FR3 of the VH to dock on six residues of *N. kaouthia* neurotoxin, while the VL domain was refractory. Four neurotoxin residues, *i.e.*, W25, C26, S31 and R 36, are located in the neurotoxin protruded long central loop (loop 2) and the other two, *i.e.*, K49 and T50, are located in loop 3. Both loops are important for high affinity binding of the neurotoxin to the α7 subunit of the neuronal acetylcholine receptor. The results of the 3D structure docking, more or less, conformed to the previous finding, which indicated by means of a phage peptide mimotope search and multiple alignments with the neurotoxin linear sequence that NkLN-HuScFv bound to the 47TVKT50 in loop 3 of the *N. kaouthia* neurotoxin [10]. Multiple alignments of the *N. kaouthia* neurotoxin amino acid sequence with neurotoxin sequences of other snakes indicated that the tentative epitopic peptide of the NkLN-HuScFv existed also in the neurotoxins of heterologous snake species, for example, 47TVKP50 and 47KVKP50 of *O. hannah* neurotoxins (accession No. AAB25587 and P01389, respectively (previous study) and 47RVNP50 (accession No. Q2VBP4 (this study)), 47TVKP50 of *Micrurus nigrocinctus* (accession No. P80548) and *N. melanoleuca* (accession No. P01383 and P01388) and 47KVKP50 of *N. oxiana* (accession No. P01427), *N.* *nivea* (accession No. P01390) and *Dendroaspis polylepis polylepis* (accession No. P01416) [10]. NkLN-HuScFv used all three CDRs of the VH to bind to seven amino acids, *i.e.*, C26, W29, G31, R33 and R36, in loop 2 and R47 and N49 in loop 3 of *O. hannah* long neurotoxin, which are important also for the acetylcholine receptor binding. Taken altogether, the results indicate that NkLN-HuScFv mediated the neurotoxin neutralization by interfering with the toxin binding to the acetylcholine receptors and, consequently, rescuing the envenomed mice from death.

The HuScFv could be prepared *in vitro* without animal immunization, and the molecule is a human protein; thus, it should be appropriate and safe for human use. The small antibodies have high solubility, reproducible refolding capacity and thermal stability, which ease the production process. It is envisaged also that a cocktail of the *in vitro* produced human ScFvs to various toxic venom components shared by different venomous snakes, exemplified by the recombinant NkLN-HuScFv in this study, should contribute to a possible future design of an improved alternative anti-snakebite remedy.

## 5. Conclusions

By using one-dimensional gel-based proteomics, the *O. hannah* holovenom was found to consist of 14 different protein types/families. *N. kaouthia* antivenin produced by the Queen Saovabah Memorial Institute (QSMI), Bangkok, cross-reacted to several functionally different *O. hannah* venom proteins and could rescue the envenomed mice from lethality. The phage display-derived recombinant *N. kaouthia* long neurotoxin-specific HuScFv cross-neutralized the *O. hannah* short and long neurotoxins and was able to extricate the *O. hannah* envenomed mice from death in a dose escalation manner. The data are useful for snakebite treatment when and where the polyspecific antivenin is not available.

## Appendixes

**Table A1:** The orthologous proteins containing peptides matched with *O. hannah* venom peptides.

Protein type/family	Protein subtype	Protein name(s)	Function(s)
1. Three-finger toxin	Long neurotoxin	OH-2, OH-4, OH-17, OH-37,OH-55 and LNTX37	These three-finger toxins bind to muscular and neuronal nicotinic acetylcholine receptors (α-7-9) and block neuromuscular transmission at the postsynaptic site causing paralysis [44,45].
	Short neurotoxin	OH-35,	This neurotoxin binds to muscarinic acetylcholine receptor and blocks synaptic nerve transmission [46].
		OH-46	This three-finger toxin binds and inhibits the nicotinic acetylcholine receptor [47].
	Cardiotoxin/Cytotoxin	CTX-15 (OH-84), CTX-21 and CTX-23	These three-finger toxins are cytotoxic and hemolytic [48].
		CTX-27 (ß Cardiotoxin)	Acts as a beta-blocker by binding to beta-1 and beta-2 adrenergic receptors. It dose-dependently decreases the heart rate. At 100 mg/kg, intraperitoneal injection into mice mediate labored breathing, impaired locomotion, lack of response to external stimuli, and death (after 30 min) [49].
	Weak neurotoxin	WNTX-34	This three-finger toxin binds to muscular and neuronal (α-7) nicotinic acetylcholine receptors with low affinity and very low affinity, respectively [46].
	Neurotoxin-like	Neurotoxin-like protein 1	Unknown
	Weak toxin	OH-DE-1	Binds to the muscular nicotinic acetylcholine receptor causes paralysis by blocking neuromuscular transmission at the postsynaptic site [38,50].
2. Phospholipase		OH-PLA_2_, OH-acidic -1 PLA_2_ and OH-acidic-2 PLA_2_ PLA_2_ Hs-1 precursor	These PLAs recognize and hydrolyze the sn-2 acyl bonds of phospholipids releasing arachidonic acid and lysophospholipids. They have neurotoxicity, anti-coagulating activity, cardiotoxicity, myonecrotic/myotoxic activity, anti-platelet activity and edema-inducing activity [35,51].
		Phospholipase B-like 1, partial	Unknown
3. Cysteine-rich secretory protein (CRiSP)		*O. hannah* opharin precursor	Binds to voltage-gated calcium channels on smooth muscle and causes high potassium-induced depolarization which weakly blocks smooth muscle contraction [52].
		Cysteine-rich venom protein-2 (Natrin-2)	This protein acts as an inflammatory modulator that interferes with wound-healing process of the bitten victim by regulating adhesion molecule expression on endothelial cells [53] and also blocks muscle contraction evoked by potassium [54].
		Cysteine-rich venom protein	Blocks contraction of smooth muscle elicited by high potassium-induced depolarization and target voltage-gated calcium channels (Cav) on smooth muscle [55].
4. Cobra venom factor/ complement C3		*O. hannah* complement-depleting factor	Anti-complement activity [56].
5. Muscarinic toxin, type A		*O. hannah* muscarinic toxin-38	This toxin binds to muscarinic acetylcholine receptor and blocks synaptic nerve transmission [57].
6. L- amino acid oxidase		L amino acid oxidase	These enzymes catalyse oxidative deamination of L-amino acid to form α-keto acid, NH_4_ and H_2_O_2_. The enzymatic activity on platelet aggregation is controversial: inhibit platelet aggregation induced by ADP and the thromboxane analog U46619 *versus* induce platelet aggregation through the hydrogen peroxide formation. The H_2_O_2_ also causes edema and human cell apoptosis [58,59,60]
7. Hypothetical protein		Hypothetical protein L345_17517 Hypothetical protein L345_07470 Hypothetical protein L345_12124 Hypothetical protein L345_15308	Unknown function
8. Low cysteine protein		Thai cobrin	Thai cobrin and ohanin (protein type/family 13, Vespryn) are classified in the same low cysteine protein family [61].
9. Phosphodiesterase-1		Phosphodiesterase-1	Phosphodiesterase catalyzes the hydrolysis of phosphodiester bonds of double-stranded and single-stranded DNA, rRNA, and tRNAs resulting in 5' mononucleotide formation and subsequent release of free adenosines which are multitoxic [62].
10. Proteases	Metalloproteinase	*Protobothrops jerdonii* disintegrin	It inhibits ADP-induced human platelet aggregation. It binds the receptor GPIIb/GPIIIa on the platelet surface resulting in hemorrhage [63].
		Zinc metalloproteinase-disintegrin-like ohanin	Snake venom zinc metalloproteinase that has hemorrhagic activity. Inhibits ADP-, TMVA- and stejnulxin-induced platelet aggregation in a dose-dependent manner (on washed platelet, but not on platelet rich plasm). Also specifically degrades alpha-chain of fibrinogen (FGA) [64]
		Zinc metalloproteinase-disintegrin-like mikarin	Snake venom zinc metalloproteinase that calcium-independently catalyzes the conversion of prothrombin (F2) to alpha-thrombin through the formation of a thrombin intermediate.[65]
		Metalloproteinase-precursor	unknown
	Kallikrein enzyme	OH fibrinogenases -α and -ß	These *O.* *hannah* venom serine proteases mediate fibrinogenolysis on both alpha and beta-chains of fibrin and amidolytic activity [66].
11. Vespryn toxin		Ohanin	Ohanin is a low cysteine protein. This neurotoxin causes dose-dependent hypolocomotion and hyperalgesia in mice. It may directly act on the central nervous system [61].
12. Kunitz	Protease inhibitor	*O. hannah* serine protease inhibitor; Protease inhibitor TCI Venom chymotrypsin inhibitor	Strongly inhibit trypsin and chymotrypsin [67].
13. Growth factor activator		Hepatocyte growth factor activator, partial Insulin-like growth factor I	Unknown function
14. Others		Coagulation factor XII, partial Endonuclease domain-containing 1 5’ nucleotidase, partial (OH)	Unknown function

**Table A2:** *O. hannah* venom components cross-reactive with horse derived *N. kaouthia* antivenin.

Spot No(s).	Protein type/family	Protein name
1 and 3	Cobra venom factor/complement C3	OVF precursor
2	Three finger toxin	Cardiotoxin-like protein
5 and 6	Cysteine rich secretory protein (CRiSP)	Ophanin/opharin and opharin precursor
7 and 10	Three finger toxin which has ancestral ten cysteine arrangements; potent in birds/reptiles [61]	Weak neurotoxin precursor
8, 9 and 10	Three finger toxin	Long chain neurotoxins
9 and 10	Three finger toxin	short chain alpha neurotoxin
4	No match	No match
